# Three-Year Longitudinal Association Between Built Environmental Factors and Decline in Older Adults’ Step Count: Gaining insights for Age-Friendly Urban Planning and Design

**DOI:** 10.3390/ijerph17124247

**Published:** 2020-06-14

**Authors:** Kimihiro Hino, Hiroyuki Usui, Masamichi Hanazato

**Affiliations:** 1 Department of Urban Engineering, Graduate School of Engineering, The University of Tokyo, Tokyo 113-8656, Japan; usui@ua.t.u-tokyo.ac.jp; 2 Center for Preventive Medical Sciences, Chiba University, Chiba 263-8522, Japan; hanazato@chiba-u.jp

**Keywords:** physical activity, neighborhood, multilevel analysis, walkability, compact city

## Abstract

This study examined the longitudinal association between the change in the step count of older adults and the neighborhood-built environment (BE) in Yokohama, Japan. We analyzed pedometer data in March 2016 and March 2019 that were acquired from 21,557 older adults aged 65–79 years at baseline, who lived in 758 neighborhoods in Yokohama City and participated in the Yokohama Walking Point Program (YWPP). Six BE variables were computed, for each of which neighborhoods were classified into quartiles. Using multilevel regression analysis, we examined the association between the BE variables, baseline step count, and change in step count. Higher population density, lower intersection density, and the second shortest quartile of the average distance to the nearest railway station were associated with a higher baseline step count. A lower intersection density and shorter average distance to the nearest railway station were associated with a smaller decline. The lowest quartile of population density was inversely associated with step-count decline. In conclusion, the neighborhood BEs were not only associated with their step count at baseline, but also widened the disparity of the step count over the three years. These findings would contribute to creating age-friendly cities where older adults can maintain and promote their health.

## 1. Introduction

The promotion of regular physical activity (PA) decreases the risk of non-communicable diseases and increases life expectancy [1]. Older adults, in particular, can benefit from regular PA to maintain their physical, social, and mental health as well as to decrease the risk of dementia [2,3]. To promote PA in a population, it is essential to achieve a walkable built environment (BE) through appropriate planning of land use and transportation [4,5]. The well-known attributes of a walkable BE, known as the “5Ds” (i.e., Density, Diversity, Design, Destination Accessibility, and Distance to Transit) [6,7], would be more impactful for older adults, as their geographic reach of activity space is smaller and they spend more time in their neighborhoods than individuals of other age groups [8,9].

Previous cross-sectional studies have examined associations between the walking behaviors and/or PA of older adults and the 5D attributes: density (e.g., population density and household density) [10,11], diversity (e.g., land-use mix and green land use) [10,12,13,14], design (e.g., intersection density and street connectivity) [6,10,11,13,14], destination accessibility (e.g., accessibility to amenities, commercial facilities, and services) [10,13,14,15], and distance to transit [16]. However, cross-sectional studies cannot avoid the self-selection bias, and longitudinal studies are needed to establish causal relationships between the BE and PA [17,18]. Thus, evidence from longitudinal studies is required to confirm the observed associations between the BE and PA that were reported from cross-sectional studies [13].

A few longitudinal studies have examined the relationship between the change of walking behaviors and/or PA of older adults and BE factors, that is, access to PA facilities [19], proximity to parks and trails [20], proximity to functional spaces (e.g., supermarkets and PA facilities) [21], proximity to services and amenities [22], residence in greener neighborhoods [23], and combined walkability score [24]. However, these studies used only some of the 5D attributes and could not consider complex interrelated BE factors in cities. Moreover, there are still few studies that use objectively measured PA data from a large sample [25], although self-serving bias cannot be avoided when using self-reported data on PA [26].

This longitudinal study aimed to examine the association between the change in older adults’ step count over a three-year period and the neighborhood BE in Yokohama, Japan. This study is unique in that it is longitudinal in nature, objectively measures step counts from a large sample, and analyzes all 5D attributes of the BE.

## 2. Materials and Methods

### 2.1. Target Area

**Figure 1:**
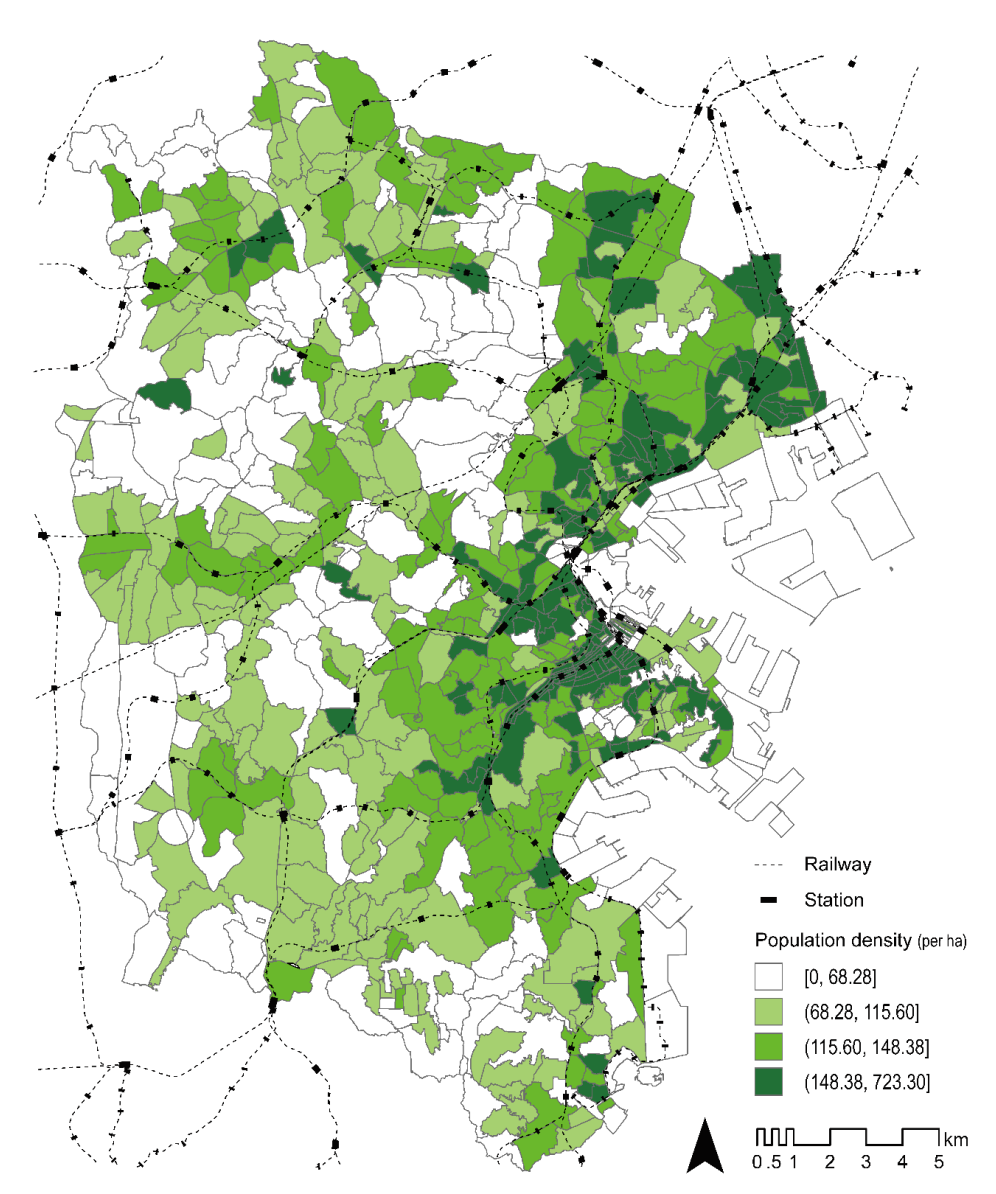
Target area (Yokohama City).

The target area of this study comprised the 758 neighborhoods (postal code areas) that constitute Yokohama City, the second most populous city in Japan with a population of approximately 3.75 million people, of whom 27.1% were in the 65 years or older age group as of January 2020. Yokohama City is one of the leading municipalities in Japan with age-friendly urban planning, design, and policies. Situated 30–40 km from Tokyo, the city’s railway network has been developed with many lines toward central Tokyo and comprises 157 railway stations (Figure 1). Similarly, as in other big cities in Japan, the railway stations in Yokohama host a comprehensive set of services and products [27] and thereby serve as a destination that is frequented by residents. The expansion of the local bus network around the railway stations ensures that approximately 90% of the citizens can access the railway stations in less than 15 min.

### 2.2. Measurement of Step Count Data

Step count data were obtained from older participants (aged 65–79 years) of the Yokohama Walking Point Program (YWPP), launched by the city authorities in November 2014 to encourage citizens to inculcate measures that could improve their health and healthy life expectancy. The program provided free pedometers (Omron HJ-326F, Japan) for adult volunteers (aged ≥18 years) who live and/or work in Yokohama. That was why we selected Yokohama City as the target area, as such large-sample data were not available in other Japanese cities. Participants were awarded points on the basis of their step count by scanning their pedometers via special readers that were installed at approximately 1000 stores and other facilities in the city. The accumulation of a certain number of points made participants eligible to win prizes. The scanned data were transferred to an online data server, and this allowed participants to monitor their step count and ranking among all of the program participants on the website [28].

We used the data obtained for March 2016 (baseline) and March, 2019 (endpoint). Yokohama has a mild climate in March, with average minimum and maximum temperatures of 7.0 and 14.5 °C in March 2016 and 7.3 and 15.1 °C in March 2019, respectively [29]. The average step count of participants in the 65 or older age group in March 2016 was the seventh highest for the preceding 12 months, starting from April 2015 [30].

**Figure 2:**
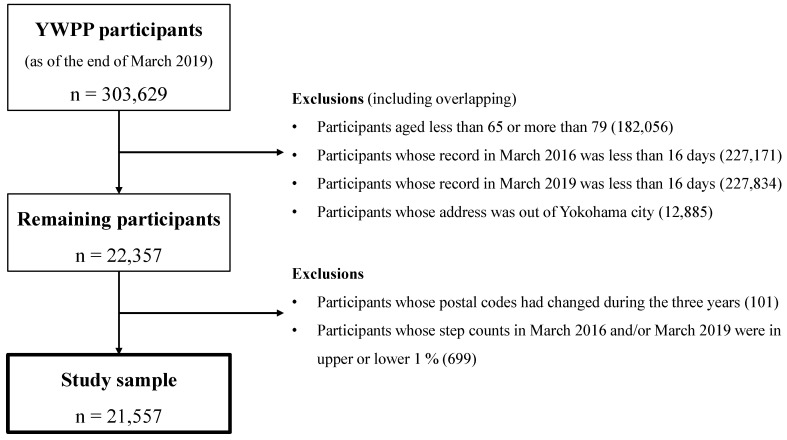
Sample selection flow diagram.

We selected the sample for the present study from among 303,629 YWPP participants (as of 31 March 2019). We excluded participants younger than 65 or older than 79 (60.0%) as well as those with a recorded participation in March 2016 or March 2019 of less than 16 days (74.8% and 75.0%, respectively). Furthermore, we excluded participants with residential addresses outside Yokohama City (4.2%), which reduced the sample to 22,357 participants. Finally, 101 participants whose postal codes had changed during the three-year period and 699 participants whose step count in March 2016 and/or March 2019 was in either the upper or lower 1% of the remaining participants were excluded as outliers. The final analysis dataset of this study included data from 21,557 participants (Figure 2).

### 2.3. Quantification of Built Environment and Control Variables

We selected six BE variables on the basis of the 5Ds that were computed using ArcMap 10.6 (Esri, Redlands, CA) and used in the subsequent analyses. Neighborhoods were classified into quartiles for each BE variable.

First, we used the population density (per ha) as an index of density (Figure 1), which we calculated from the population census conducted in 2015. Second, we used the intersection density (per ha) as an index of design, as previously reported [6,10,11]. The Advanced Digital Road Map Database 2013 (Sumitomo Electric Industries, Ltd., Osaka, Japan) was the source of information. Third, we used the proportion of commercial land use (%) as an index of diversity and destination that was calculated from the result of the Basic Surveys Concerning City Planning in 2013 conducted by Yokohama City. Fourth, we employed the normalized difference vegetation index (NDVI), from 2016, as another index of diversity and destination. The NDVI quantifies vegetation by measuring the difference between near-infrared light (which vegetation strongly reflects) and red light (which vegetation absorbs) and ranges from −1 to +1. A positive NDVI indicates that the land cover is likely to be green vegetation, including parks and promenades, which can be potential recreational destinations for residents. However, a negative NDVI indicates that the land cover is likely to be street surfaces and buildings. The NDVI was selected as a BE variable because green land use was associated with the PA of older adults in both cross-sectional [31] and longitudinal studies [23]. Fifth, the average distance to the nearest railway station (km) was used as an index of the distance to transit and destination, and was calculated from the data of the National Land Numerical Information download service obtained in 2018 [32]. Last, the average distance to the nearest bus stop (km) was used as an index of the distance to transit, which was calculated from the data of the same service obtained in 2010. Given the shared role between railways and buses in Yokohama, as mentioned earlier, the average distance to the nearest railway station and bus stop should be separate variables; however, most previous studies did not separate the two [14,33]. As the precise address of each participant was unknown, the distance to the nearest railway station and bus stop from each participant’s address was computed by measuring the distance from the 50-m grid points within each neighborhood to calculate the average for each neighborhood.

Many studies have used a composite measure of walkability [16,24,34,35,36,37]. However, in this study, we used BE variables without compounding them, because composite measures have less utility for urban planners and designers as they cannot ascertain the effect of each BE variable. The control variables in both of the analyses included the sex and age group (65–69, 70–74, and 75–79 years, as of March 2016) of the participants.

### 2.4. Statistical Analysis

As our data had a multilevel structure with individuals (Level One) nested within 758 neighborhoods (Level Two), we conducted multilevel regression analyses with random intercepts. The baseline step count and the change in step count during the three-year period were the outcome variables for the first and second analyses, respectively. The descriptive variables comprised the six BE variables and control variables of sex and age groups in the first analysis, and the baseline step count was included in the second analysis. The fourth quartile of six BE variables, female sex, and age of 75–79 years were set as the reference category. The significance level was set at *p* < 0.05. All statistical analyses were conducted in IBM SPSS Statistics 26 (IBM Corp., Armonk, NY, USA).

## 3. Results

### 3.1. Descriptive Statistics

Table 1 presents the descriptive statistics of the study sample. Males constituted 48% of the sample. The age groups 65–69, 70–74, and 75–79 constituted 43%, 35%, and 22% of the sample, respectively. The average step count at the baseline was 7699.0 ± 3252.1, and the count at the endpoint was 6845.3 ± 3252.1, which indicated that the step count of the sample decreased by 850 steps, on average, during the three-year study period.

Descriptive statistics of the BE variables of 758 neighborhoods are presented in Table 2. The median values of population density and intersection density per ha were 115.60 and 2.03, respectively. The proportion of commercial land use was generally low, with a median proportion of 3.3%. The NDVI was positive in all neighborhoods. The median of the average distance to the nearest railway station and the nearest bus stop was 647 and 175 m, respectively, which represents the convenience of public transportation in Yokohama.

### 3.2. Multilevel Regression Analyses

The results of the multilevel regression analyses are presented in Table 3. In the first analysis, with the outcome variable of the baseline step count, a higher population density and lower intersection density were associated with a higher baseline step count. The association between the average distance to the nearest railway station and baseline step count was nonlinear; however, the second shortest quartile (Q2) was associated with a significantly higher baseline step count. Other BE variables, such as the proportion of commercial land use, the NDVI, and the average distance to the nearest bus stop, did not show significant associations.

In the second analysis with the outcome variable of change in step count during the three years, the lowest quartile (Q1) of population density was associated with a larger decline in the step count. In addition, a lower intersection density and shorter average distance to the nearest railway station were associated with a smaller decline in step count. However, the other three BE variables did not have significant associations.

With regard to control variables over the course of the study, female sex and older age were associated with a lower baseline step count and larger decline in the step count. Moreover, a higher baseline step count was associated with a larger decline.

## 4. Discussion

This study examined the association between the change in the step count of older adults and the neighborhood BE in Yokohama, Japan, over a three-year period. The main finding was that four of the 5Ds (Density, Design, Destination Accessibility, and Distance to Transit) were associated with a decline in older adults’ step count. In addition, the association of the BE with baseline step count was examined. Comparisons of the results of the two models showed that the significant variables selected were mostly the same, and the direction of the coefficients was consistent in both models. Thus, the BEs of older adults were not only associated with their step count at a certain timepoint, but also widened the disparity of their step count over the three years.

With regard to each variable, a lower population density was associated with a lower baseline step count, and older adults living in the lowest quartile neighborhoods of population density had a significant decrease in the step count during the three years. Population density ensures public transport and local shops and services are more viable [38] and serves as a proxy variable showing the convenience of walking for local residents. Accordingly, our result agrees with that of a three-year study in Québec, Canada, that proximity to local shops and services was associated with greater likelihood of frequent walking [22]. Cross-sectional studies on older Japanese adults that found an association between population density and their body mass index [39] and physical function [40] underpin our result. On the other hand, older adults in sparsely-populated and inconvenient areas might decrease their trip frequency or change their transportation mode from walking. It was estimated that the population of Yokohama reached its peak in 2019 and would begin declining as with other cities in Japan [41]. On a microscale level, areas that are inconvenient to reach in the city would experience a greater decrease in population, resulting in the withdrawal of facilities and even greater inconvenience to residents. Considering low residential mobility in Japan, especially that of older adults [42], health promotion interventions for PA would be necessary for such areas. Deployment planning of facilities that serve as third places to which older adults routinely walk and where they can socialize with others, would be important [43,44].

Inversely, lower intersection density was associated with higher baseline step count and a smaller decline in step count over the course of the study. High intersection density indicates that there are many route opportunities for traversing a road network and is thought to promote walking [6], and previous cross-sectional studies have shown that there is a positive association between older adults’ self-reported walking and intersection density [10,11]. In previous longitudinal studies, not limited to older adults, higher street connectivity proved to be associated with a greater increase in walking for transportation [45]. Although our results contradicted these studies, another study conducted in Japan stated that the number of intersections was negatively associated with walking time [46]. It may be that intersection density cannot be an index of walkability in a city with high intersection density, such as Yokohama. The intersection density in Yokohama is approximately twice this figure compared to 100 cities all over the world, excluding Japanese cities, and as high as the highest city (Lisbon, Portugal) in developed countries [47]. Considering a previous study that showed that the number of street intersections contributed to walking activity only when residents perceived that the traffic conditions were safe [11], older adults in neighborhoods with a high intersection density in this study might feel unsafe, as the intersection density acted as an index of traffic accident risk. The installation of more traffic light buttons for people who walk slowly, especially older pedestrians, should be considered for their safety.

With regard to the average distance to the nearest railway station, older adults living in the second closest quartile neighborhood recorded a significantly higher baseline step count, which suggests that they could not walk much when living too close to stations. A walkable design where older adults feel safe and are more willing to walk around is desirable around railway stations. More importantly, it was found that older adults living closer to railway stations experienced a smaller decline in step count. Taking this into consideration with a previous study of older Japanese that showed a lower risk of functional limitations among those using public transport after driving cessation [48], closeness to railway stations would enable older adults to maintain their active living, their step count, and their health even after they give up driving. Yokohama City aims to accumulate residential functions around railway stations, even in suburban areas, as well as commercial and business functions [49]. Such a compact city policy, which is thought to positively influence the overall health of city populations [50], would be more beneficial in an aging society such as Yokohama City, provided that consideration is given to outskirt areas, as noted earlier.

The other BE variables, including the proportion of commercial land use, the NDVI, and the average distance to the nearest bus stop, were not significantly associated with step counts in either regression analyses. This inconsistency with a previous study in Norfolk, United Kingdom, which reported that neighborhood greenspace may be protective against a decline in older people’s PA [23], seemed to be due to the difference of the degree of urbanization. With regard to personal attributes, females and participants in the older group recorded a lower baseline step count and a larger decline over the course of the study. Furthermore, a higher baseline step count was associated with a larger decline, which was consistent with the results from a previous study [19]. Similar results are expected in other Japanese megacities with high population density and developed railway network (e.g., Tokyo, Osaka, and Nagoya); however, it should be reexamined when pedometer data from as large a sample as this study are obtained in other cities.

This study makes an important contribution to existing research on BE factors that mitigate the decline in the step count of older adults, although it has some limitations. For instance, previous studies showed social environmental factors, such as neighborhood cohesion [51], income deprivation [52], participation with friends [53], social support [35], and neighbor relationships [54], were associated with walking behaviors and/or PA. Unfortunately, this study was not able to assess the impact of social environments of older adults as it is difficult to collect data on social environmental factors from a large sample. Therefore, future studies should consider them when examining the longitudinal association between changes in the PA of older adults. In addition, study participants may not be representative of the general population of Yokohama City in that they were motivated to have pedometers, suggesting a greater intention to walk than others, although approximately 20% of citizens in the age group of 65–79 participated in the program. We thought that study participants would be less affected by the BE because of their higher motivation and that the effect of the BE would be underestimated in this study.

## 5. Conclusions

This three-year study examined the longitudinal association between the change in step count of 21,557 older adults and the neighborhood BEs in Yokohama, Japan, using multilevel regression analysis. The main finding of this study was that the neighborhood BEs of older adults were not only associated with their step count at a certain time but also widened the disparity in the step count over the three years, which had not been examined in previous cross-sectional studies or those using self-reported data on PA. It is essential that urban planners and designers create compact cities located around railway stations that are protected from traffic to ensure the safety of older adults. Our findings, providing insights into the relationship between neighborhood BEs and health behavior of older adults, could contribute to creating such age-friendly cities where older adults can maintain and promote their health.

## Figures and Tables

**Table 1:** Descriptive statistics of the sample (*N* = 21,557).

Category		*N*	%
Sex	Male	10,303	47.8
	Female	11,254	52.2
Age (in years)	65–69	9359	43.4
	70–74	7507	34.8
	79–79	4691	21.8

**Table 2:** Descriptive statistics of built environment (BE) variables of 758 neighborhoods.

Category	Min	Max	Mean	SD	1st Quartile	Median	3rd Quartile
Population density (per ha)	0	723.30	116.66	79.11	68.28	115.60	148.38
Intersection density (per ha)	0	7.72	2.09	1.18	1.33	2.03	2.76
Proportion of commercial land use (%)	0	51.18	5.99	7.56	1.54	3.30	6.76
NDVI	0.01	0.34	0.08	0.05	0.05	0.08	0.10
Average distance to the nearest railway station (km)	0.07	3.99	0.82	0.62	0.36	0.65	1.06
Bus stop (km)	0.06	1.72	0.20	0.10	0.14	0.18	0.23

SD, Standard Deviation; NDVI, Normalized Difference Vegetation Index.

**Table 3:** Associations between BE variables and step count (baseline and change) using multilevel regression (*N* = 21,557).

	Baseline Step Count			Change in Step Count		
Category	*B*	95 % CI	*p*	*B*	95 % CI	*p*
BE variables						
Population density (ref: Q4)						
Q1 (Lowest)	−213.7	(−396, −31.3)	0.022 *	−115.9	(−225.9, −5.9)	0.039 *
Q2	−118.9	(−262.1, 24.4)	0.104	−10.8	(−97.6, 76)	0.807
Q3	−113.3	(−250.4, 23.7)	0.105	−4.3	(−87.4, 78.8)	0.919
Intersection density (ref: Q4)						
Q1 (Lowest)	208.0	(52.5, 363.4)	0.009 **	87.9	(−5.4, 181.3)	0.065
Q2	50.7	(−75.6, 177)	0.430	129.7	(53.3, 206.2)	0.001 ***
Q3	−37.0	(−157.4, 83.4)	0.545	31.9	(−41, 104.8)	0.391
Average distance to the nearest railway station (ref: Q4)						
Q1 (Shortest)	120.5	(−49.2, 290.2)	0.163	130.7	(27.9, 233.5)	0.013 *
Q2	200.0	(73.1, 326.9)	0.002 **	117.5	(40.7, 194.3)	0.003 **
Q3	110.0	(−3.8, 223.8)	0.058	117.1	(48.6, 185.6)	0.001 ***
Control variables						
Sex (ref: Female)						
Male	1821.7	(1738.4, 1905)	0.000 ***	358.6	(304.3, 412.8)	0.000 ***
Age (ref: 75–79)						
65–69	815.4	(705.8, 925)	0.000 ***	314.9	(246.1, 383.8)	0.000 ***
70–74	460.7	(347, 574.4)	0.000 ***	154.0	(82.7, 225.2)	0.000 ***
Baseline step counts	–	–	–	−0.3	(−0.3, −0.3)	0.000 ***

BE, built environment; *B*: regression coefficient; CI, confidence intervals; *p*: statistical significance of coefficient (* < 0.05, ** < 0.01, *** < 0.001).

## Data Availability

The data that support the findings of this study are able to be obtained from Yokohama City, but restrictions apply to the availability of these data. They were used under an agreement for the current study, and so are not publicly available.
